# Environmental and Occupational Risk Factors Associated with Chronic Kidney Disease of Unknown Etiology in West Javanese Rice Farmers, Indonesia

**DOI:** 10.3390/ijerph17124521

**Published:** 2020-06-23

**Authors:** Laila Fitria, Nurhayati Adnan Prihartono, Doni Hikmat Ramdhan, Tri Yunis Miko Wahyono, Pornpimol Kongtip, Susan Woskie

**Affiliations:** 1 Department of Environmental Health, Faculty of Public Health, University of Indonesia, Depok 16424, Indonesia; 2 Department of Epidemiology, Faculty of Public Health, University of Indonesia, Depok 16424, Indonesia; nurhayati-a@ui.ac.id (N.A.P.); triyunis@yahoo.com (T.Y.M.W.); 3 Department of Occupational Health and Safety, Faculty of Public Health, University of Indonesia, Depok 16424, Indonesia; doni@ui.ac.id; 4 Department of Occupational Health and Safety, Faculty of Public Health, Mahidol University, 420/1 Rajvidhi Road, Bangkok 10400, Thailand; pornpimol.kon@mahidol.ac.th; 5 Department of Public Health, University of Massachusetts Lowell, One University Ave, Lowell, MA 01854-2867, USA; Susan_Woskie@uml.edu

**Keywords:** CKD, CKDu, male farmer, environmental factor, occupational factor

## Abstract

Chronic kidney disease of unknown etiology (CKDu) in agricultural population is an increasing issue. This study aims to obtain a prevalence estimate of CKDu in male rice farmers in West Java, Indonesia; and analyze the relationship between CKDu and environmental and occupational factors. The study design was cross-sectional. This study included 354 healthy male farmers in two rice agriculture areas in West Java with different altitudes (low altitude and high-altitude location). This research included blood and urine sampling from the farmers for serum creatinine (SCr) level and proteinuria; questionnaire on demographic information, occupational factors and other risk factors for CKDu. We measured ambient temperature and humidity in both study locations for environmental factors. From SCr level and proteinuria, we categorized the farmers into five stages of CKD; then we distinguished CKDu from CKD if the farmers had stage 1–4 of CKD but without diabetes, hypertension and other traditional causes. Data were analyzed with multivariate logistic regression to get prevalence odd ratios of CKDu and its relationship with environmental and occupational factors, adjusted with other risk factors of CKDu. The overall prevalence of CKD was 24.9% and CKDu was 18.6%. For the environmental factors, farm location (high altitude versus low altitude location) was associated with CKDu (Prevalence Odds Ratio (POR): 2.0; 95% CI: 1.2–3.5). For the occupational factors, although not significant, the risk of CKDu increased with the longer use of insecticide and with the more frequent of insecticide use. We suggested that there was a need to conduct future research to investigate more on the association of those environmental and occupational factors with CKDu.

## 1. Introduction

Chronic kidney disease (CKD) is a progressive condition marked by the gradual loss of kidney function. According to 2019 United State Renal Data System (USRDS) data, the most common traditional causes of CKD are diabetes, hypertension, glomerulonephritis and cystic kidney [1]. While in all developed and many developing countries, diabetes and hypertension are the leading causes of CKD, glomerulonephritis and unknown causes are more common in countries of Asia and sub-Saharan Africa [2]. Recent interest in chronic kidney disease of non-traditional causes, also named as chronic kidney disease of unknown etiology (CKDu) has identified both individual and environmental/occupational risk factors. CKDu distinguished from CKD, if a person had low eGFR (<60 mL/min/1.73 m^2^) and proteinuria, but without having diabetes, hypertension, kidney stone, urethritis and gout [3]. Excessive fructose intake, alcohol use and smoking are known as individual risk factors that directly associated with CKDu [4]. Environmental risk factors associated with CKDu are agrochemical exposure (pesticides and fertilizers), heavy metal exposure (cadmium, arsenic) from food and water consumed and chronic dehydration [3].

Studies showed the prevalence of CKD Stage 1 to 5 ranges from 8.66% (South Africa, Senegal, Congo) to 18.38% (Europe) [5]. In Malaysia, the prevalence of chronic kidney disease was 9.07% [6]. In Indonesia, there was no definitive data on the prevalence of CKD, but The Basic Health Research (Riskesdas) showed 0.2% of the population in Indonesia reported having CKD in 2013 [7]. The treatment of CKD is costly, it is progressive and requires hemodialysis or renal transplant to prevent death in the late stages. In 2017, 30,831 new hemodialysis patients were registered in Indonesia, of these, more than 48.3% were associated with hypertension or diabetes and 4% were identified as having unknown etiology [8].

Several studies have shown an association between CKDu and agricultural activities, especially in agricultural lands where agrochemicals are used intensively [9,10,11]. Agrochemicals are known to have harmful health effects on humans, including renal damage and there are over 1000 active compounds sold as agrochemicals, including pesticides and fertilizers [11]. To study the relationship between each active compound and CKDu is thought to be impractical. A study by Valcke et al. in 2017 concluded that existing studies provide scarce evidence for an association between pesticides and regional CKDu epidemics, but, given the poor pesticide exposure assessment in the majority, the role of nephrotoxic agrochemicals cannot be conclusively discarded [11]. The study also recommended that future research should procure an assessment of lifetime exposures to relevant specific pesticides.

Indonesia is an agrarian country where about 40 percent of the population are farmers scattered throughout all Indonesian provinces. West Java, Central Java and East Java are three provinces with the highest number of farmers, 105,677 households of farmers in West Java, 94,689 households of farmers in Central Java and 211,700 households of farmers in East Java [12]. As an agrarian country, the use of pesticides in agriculture is quite high in Indonesia [13]. The Ministry of Agriculture has reported an increase in the use of agricultural pesticides from 2008 to 2014. Among the types of pesticides in use, insecticides and herbicides were most widely used in Indonesia (1198 brands of insecticides and 944 brands of herbicides) [13].

As a country located at the equator, Indonesia is also experiencing the effects of global warming. In the long term (1900–2010) temperature rise in Indonesia amounted to 0.002 °C per year, over the recent period of 50 years (1965–2009) the increase in temperature was even faster (0.016 °C per year) [14]. With increased temperature from climate change, it has been suggested that the risk of heat-related injuries and illnesses among workers closer to the equator will increase [15]. Studies have shown that altitude also plays important role in CKDu, where farmers in low altitude (hotter and more humid) areas were at higher risk of CKDu compared to farmers in higher altitude areas (cooler and less humid) [4].

To date, no study has been conducted to investigate the prevalence of CKDu and its risk factors in the agricultural population in Indonesia. We found some studies on CKD in Indonesia, but not CKDu and these studies focused only on subjects already diagnosed with kidney disease [16,17,18,19,20]. As unknown causes in CKD are more common in countries of Asia [2], we interested to conduct a study obtaining a prevalence estimate of CKDu in farmers in Indonesia and its risk factors.

This study aims to: (a) obtain prevalence estimate of CKDu in male rice farmers in West Java, Indonesia; (b) analyze the relationship between CKDu and environmental and occupational factors.

## 2. Materials and Methods

### 2.1. Ethical Clearance

This research was reviewed by The Research and Ethics Committee of Faculty of Public Health, University of Indonesia (letternumber 73/UN2.F10/PPM.00.02/2018, 6 March 2018). Study participants were fully informed about the study protocol and all participants signed their written informed consent to participate in this study.

### 2.2. Study Design

Rice farming is one of the biggest agriculture especially in West Java, Indonesia. The male farmers are the main workers involved in rice farming. Hence, in this study, we focused on male rice farmers in West Java.

We conducted a cross-sectional survey among male rice farmers in two different geographic locations; Karawang and Bogor Regency, West Java Province. Karawang is known as the largest rice paddy area in Indonesia and is located 2 hours from Jakarta. Karawang Regency is located in a low altitude region at about 0–5 m above sea level [21]. While Bogor Regency also produces rice and is located in a high-altitude region at about 450 m above sea level, 3 hours from Jakarta [22]. From each regency, we selected one village which represented the greatest difference in altitude, and which had a large farmer population: Sukamerta Village in Karawang Regency and Jambuluwuk Village in Bogor Regency.

In this research, we had followed the STrengthening the Reporting of OBservational studies in Epidemiology (STROBE) criteria for cross-sectional studies [23].

### 2.3. Population and Sample

The study population was adult male rice farmers between the ages of 20 through 65 years who currently live in the villages. A person was defined as being a resident of Karawang and Bogor Regency if he lived there for at least the previous 2 years. Male rice farmers were chosen as the target population in this study because male farmers work in the field each day and they usually do the pesticide application in the field.

We used the household list from the village office (in Karawang) and the farmers list from the head of the farmers’ group (in Bogor) as the sampling frame to recruit the study subjects (male farmers) using a systematic random sampling method. By using sample size formula for proportion estimation, we calculated the required sample size based on an estimated prevalence of CKD was 9% [6], 95% confidence level and estimated precision was 0.05, and we estimated a required sample size of 126 subjects in each village. Because of possible blood sample damage or lysis and nonparticipation, we selected 200 subjects in each village or a total of 400 subjects.

### 2.4. Data Collection Procedures

Three teams were involved in data collection in each village, and they worked at the same time. One team consisted of five interviewers who went house-to-house to recruit study participants and collect data using a questionnaire; another team consisted of laboratory staff who collected blood and urine samples, anthropometry and blood pressure measurements from participants; and a third environmental team measured ambient temperature and relative humidity, as well as air velocity in the rice field.

Before the data collection, a copy of the script for obtaining verbal permission, which briefly explains the reason for the study, was read to the potential subjects. After signing the informed consent form, the subject was then administered a questionnaire on demographic and risk factors for CKD and CKDu, such as age, nutritional status, education levels, current diseases related with CKD (hypertension, diabetes mellitus) as recommended in the DEGREE Core Protocol [24]. In addition, we obtained information on the water sources for drinking. Concerning occupational factors as a farmer, we obtained information on years of work as a farmer, type of farming and pesticide use (the type of pesticides and methods of pesticide application, use of personal protective equipment and pesticide use at home). For the questionnaire used in this study, no reliability testing was done because the source of the original questionnaire was from DEGREE which was based on WHO Steps protocol for Non-communicable Disease (NCD) surveillance [25,26].

At the end of the week, all subjects enrolled in the study were gathered at the village office and had a venous blood sample drawn. This was performed by a phlebotomist of PRODIA Laboratory, which is a leading clinical laboratory in Indonesia and has fulfilled the requirements of ISO 9001 2008 and ISO 15189 [27]. We used the standard procedures for collecting the venous blood and urine samples. From blood samples, we measured serum creatinine (by Colorimetry Enzymatic method) [28], urea (by Enzymatic UV test, urease–GLDH) [29] and glucose (by Hexokinase method, using automated clinical analyzer TMS 24i Premium (Tokyo Boeki Medisys Inc. Tokyo, Japan), with Proline reagent). From the urine, glucose and protein were measured by the Reflectance Photometry method, using semi-automated urine analyzer Cobas u411, with Combur 10 Test M reagent. Moreover, all study subjects also had physical measurements, i.e., blood pressure and anthropometry (weight and height).

We measured the working environment conditions everyday during the whole week of the data collection. We used 3M^™^ QUESTempo^™^ 34 thermal environmental monitor (wet ball temperature, dry ball temperature, globe temperature, relative humidity [RH] and absolute humidity) to measure ambient temperature and relative humidity. wet bulb globe temperature (WBGT) was recorded every hour for the entire working day in the rice fields, started at 6 am to 6 pm. The air velocity was measured using a digital anemometer.

### 2.5. Data Processing

CKD was determined using several metrics, i.e., serum creatinine (SCr) level, proteinuria and glomerular filtration rate (eGFR), which calculated using the Chronic Kidney Disease - Modification of Diet in Renal Disease (CKD–MDRD) equation [30]. We used the term “proteinuria” instead of “albuminuria” in our study, because of the urine test that we used in our study (Combur 10 Test M Strips) detected proteinuria and could not measure albuminuria accurately [31].

We categorized study subjects into categories for each parameter. For SCr level, subjects with SCr level >1.2 mg/dL were categorized as having high serum creatinine, and those with SCr level ≤1.2 mg/dL were categorized to have normal serum creatinine. For proteinuria, study subjects with +2 or more for protein category on dipstick were categorized into positive proteinuria, and while if study subjects had 0 or +1 on the dipstick for protein were categorized as negative. Moreover, finally for eGFR, study subjects with eGFR <60 mL/min/1.73 m^2^ were categorized as low eGFR, and while subjects with eGFR ≥60 mL/min/1.73 m^2^ were categorized as high eGFR.

For CKD categorization, first we categorized study subjects into five stages of CKD. Normal or no CKD (eGFR ≥ 90 mL/min/1.73 m^2^ and negative proteinuria); Stage 1 CKD (eGFR >90 mL/min per 1.73 m^2^ and positive proteinuria); Stage 2 CKD (eGFR 60–89 mL/min per 1.73 m^2^ and positive proteinuria); Stage 3 CKD (eGFR 30–59 mL/min per 1.73 m^2^); Stage 4 CKD (eGFR 15–29 mL/min per 1.73 m^2^); and Stage 5 CKD (eGFR >15 mL/min per 1.73 m^2^) [2]. From the data obtained, there were no study subject identified in the Stage 5 CKD category, because based on our measurement there were no study subject that had eGFR lower than 15 mL/min per 1.73 m^2^.

Then for further analysis, we categorized study subjects into three categories based on those CKD stages: No CKD (normal); CKD, if the study subject had Stage 1 to Stage 4 CKD; and CKDu, if the study subject had Stage 1 to Stage 4 CKD, but without diabetes based on blood sugar concentration (>200 mg/dL) or self-reported diabetes, no hypertension based on blood pressure (systole/diastole ≥140/ ≥90) or self-reported hypertension, but using medication [4]; these CKDu subjects also had to have no self-reported urethritis, gout or kidney stones. In our analysis, we separated CKDu from CKD to obtain the prevalence of both CKD and CKDu in male rice farmers, and to find potential risk factors for CKDu in our study areas.

To find risk factors for CKD and CKDu in our study areas, we distributed the prevalence of CKD and CKDu by location, socio demographic factors, and several risk factors including pesticide use. For the location, we set Karawang Regency as ‘low altitude and high WBGT’ location and Bogor Regency as ‘high altitude and low WBGT’ location. This was based on the fact that Karawang Regency is located in a low altitude region (at 0–5 m above sea level) and Bogor Regency is located in high altitude region (at 450 m above sea level). In addition, our measurements of working environment conditions consistently showed that low altitude Karawang had higher WBGT temperatures than Bogor [32].

For pesticide use, we adopted a semi-quantitative method to estimate long-term pesticide exposures [33]. Pesticide exposure data were gathered from the questionnaire (including questions about mixing and application of pesticide, repairing pesticide spray machine and the use of personal protection equipment when applying pesticide in the field) and then scored, and the scores were calculated as Intensity Level using the general algorithm by Dosemeci et al [33]. Insecticides were used by all farmers who used pesticides in farming, while herbicides and fungicides were not commonly used in our study area. For this reason, we focused on insecticides for developing the exposure estimates. Fifteen percent of farmers used only one brand of insecticide, while 23 percent used two brands and 21 percent used three brands of insecticide in our study area. For this reason, we calculated the Intensity Level using only the most used insecticide by each farmer. We also collected information on insecticide use in home: by spraying, using coil or study form of insecticide, electric coil, etc.

### 2.6. Statistical Analysis

We applied Multivariate Logistic Regression Analysis with the CKDu as the dependent variable, to assess potential risk factors for CKDu in male rice farmers in our study areas, and also to get prevalence odd ratios of CKDu associated with the environmental factor (altitude of farm location) and occupational factor (pesticide exposure), adjusted with other risk factors of CKDu (age, years of work as a farmer, type of farming, hours work as farmer per day, body mass index, kidney stone, urethritis, family history of kidney disease, herbs consumption, alcohol consumption, smoking, drug consumption, water source for drinking and insecticide use at home). We excluded study subjects with CKD in this analysis because we intended to focus on observing the relationship between CKDu and environmental/occupational factors.

## 3. Results

From 400 targeted farmers, 354 farmers agreed to participate in the whole process of the data collection (186 farmers in Karawang and 168 farmers in Bogor), giving a response rate of 88.5%. However, based on the required sample size calculation before (which was 126 subjects in each location), the requiredsample size was fulfilled.

Table 1 shows the sociodemographic characteristics of the male farmers in the two study locations. Farmers in Karawang and Bogor were relatively at the same age, but the farmers’ educational level in Karawang was slightly higher compared to Bogor farmers. Farmers in Bogor are more likely to have a higher prevalence of hypertension based on their answer in the interview, but blood pressure measurement shows different results, that more farmers in Karawang had high blood pressure (≥140/ ≥90) compared to farmers in Bogor. Farmers in Karawang also had higher blood sugar based on blood sugar measurement (>200 mg/dL) and a higher prevalence of diabetes mellitus based on their answers in the interview.

As seen in Table 2, the prevalence of CKD and CKDu and all CKD parameters, were consistently higher in Bogor than in Karawang. In Bogor, study subjects with CKD (Stage 1–4) were 12.7% higher compared to Karawang. This calculation of CKD prevalence included all study subjects, whether they had diabetes or hypertension. But for the calculation of CKDu, we excluded study subjects that had CKD, but suffered from diabetes or hypertension (both based on measurement or self-report). From the calculation for CKDu prevalence, we obtained 9.8% more study subjects in Bogor that had CKDu compared to Karawang.

In Table 3, we distributed the farmers according to CKD status (CKDu, CKD and No CKD or Normal), individual factors, occupational factors and environmental factors. The prevalence of CKDu was slightly higher in older aged farmers, whereas CKD prevalence was higher in younger aged farmers. Among study subjects who had worked longer as a farmer (more than 20 years), the prevalence of both CKDu and CKD was higher compared to study subjects who had worked 15–19 years. But interestingly, among farmers who worked shorter (less than 15 years), the prevalence of CKDu and CKD was also higher compared to those who worked longer (15–19 years). As we compared CKDu prevalence according to the type of farming, we could see that more farmers who used traditional practices in farming had CKDu than farmers who used mixed or modern farming practices. Traditional farming practices are intensively manual including cultivating land with hoes, harvesting rice with sickles, etc. Modern methods of farming use tractors for cultivating and harvesting, while mixed methods use some of both.

We also compared the prevalence of CKDu and CKD by consumption of traditional drinks and alcohol, as well as smoking. The prevalence of CKD was higher in farmers who consumed traditional drinks, but the prevalence of CKDu was not so different. Traditional drinks commonly consumed by farmers in our study area included homemade and factory-made herbal drinks or herbal medicines. Alcohol consumption was not common among farmers, but almost all farmers were smokers. We could not see a difference in CKDu and CKD prevalence among farmers who were current smokers.

We got interesting findings when we compared the prevalence of CKDu and CKD according to the water source for drinking. The highest prevalence of both CKDu and CKD was among farmers who used surface water (including water from pond and river) for daily drinking. CKDu and CKD prevalence were slightly lower among farmers who used ground well or spring water for drinking and the lowest prevalence was among farmers who used government piped water or bottled water for drinking.

We compared CKDu and CKD prevalence according to pesticide use in farming and at home (Table 4). Almost all farmers used pesticides in farming and surprisingly both prevalence of CKDu and CKD was higher among farmers who did not use pesticides, although they were represented by small numbers. The highest prevalence of CKDu was among farmers who had used insecticide for less than 10 years, while the highest prevalence of CKD was among farmers who had used insecticide for 10 to 19 years. The highest prevalence of both CKDu and CKD was also found in farmers who most rarely used insecticide compared to farmers that more often in insecticide use. We calculated the intensity score of the most common current insecticide used, which resulted in a range of 0 to 21 with a median of 16. We compared the prevalence of CKDu and CKD according to the intensity score of current insecticide use, and we found a higher prevalence of both CKDu and CKD among farmers with a higher intensity score for insecticide exposure. We obtained data about insecticide use at home. The data showed that the prevalence of CKD was higher in farmers who used insecticide at home, but the prevalence of CKDu was higher in farmers who did not use insecticide at home.

We used multivariate logistic regression to examine the association of CKDu with a range of potential risk factors (age, years of work as farmer, type of farming, hours work as farmer per day, BMI, kidney stone, urethritis, family history of kidney disease, herbs consumption, alcohol consumption, smoking, drug consumption, water source for drinking, farm location and pesticide use). Our findings showed that the risk factor for CKDu was farm location (POR 2.0; 95% CI 1.2–3.5), which was farmers in high altitude/low WBGT location (i.e., Bogor) were at two times higher risk for having CKDu compared with farmers in low altitude/high WBGT location (i.e., Karawang). These findings were not in line with our initial hypothesis, that farmers in low altitude/high WBGT locations would have a higher risk for CKDu. To explain these findings, we carried out stratification analysis with variables that potentially could make any difference between the two locations, i.e., type of farming, as shown in Table 5. From the results of the analysis, we could see that among farmers who applied less mechanized farming (traditional or mixed farming method), the risk for having CKDu was higher in high altitude/low WBGT locations. Among farmers who applied mixed type of farming, the risk for having CKDu was 2.9 times higher in high altitude/low WBGT locations (95% CI 1.3–6.4). Although not significant, the risk for having CKDu was also higher in high altitude/low WBGT locations among farmers who applied traditional type of farming. But in contrary, although not significant, the risk for having CKDu was lower in high altitude/low WBGT locations among farmers who applied modern type of farming.

We estimated the risk of CKDu associated with insecticide exposure after controlling for farm location and other covariates (Table 6). The risk of CKDu increased along with the years of insecticide use and frequency although the findings were not statistically significant. This result indicated that potential risk factors of CKDu may not be limited to the exposure of insecticides.

## 4. Discussion

To our knowledge, our study is the first in Indonesia to study CKD and CKDu prevalence in a general working population, i.e., among farmers that are still actively working. Most of the CKD studies in Indonesia have focused on subjects already diagnosed with kidney disease [16,17,18,19,20]. We used common diagnostic criteria for CKD, similar to other studies of working populations in El Salvador [3] and Malaysia [6]. For our CKDu criteria, we used exclusion criteria that matched a study of sugarcane workers in El Salvador [3].

The prevalence of CKD among male farmers in our study was 24.9%, which is similar to the findings among sugarcane workers in El Salvador—20.2–28.9% [3]. The prevalence of CKDu in our study location was 18.6%, which was similar to a study in Sri Lanka—15.1–22.9% [34]. We could not compare the prevalence of CKD in other areas because of differences in the criteria used to identify cases of CKD and CKDu.

Our findings showed that one of the environmental risk factors for CKDu was farm location. Bogor Regency was located in high altitude (above 450 m) and had lower WBGT temperatures. While Karawang Regency was located in a lower altitude (0–5 m) and had a high WBGT temperature. Farmers in Bogor Regency were at two times higher risk for having CKDu compared farmers in Karawang Regency. This finding is the opposite of our original hypothesis which was based on a study in El Salvador that showed sugarcane workers who lived and worked in coastal areas (low altitude higher temperature environment) had elevated SCr levels and lower eGFR compared with sugarcane workers in communities above 500 m [3].

To explain these findings, we carried out stratification analysis with variables that potentially could explain the difference between the two locations, i.e., type of farming. The analysis showed that among farmers who applied a modern type of farming which is much more mechanized, the risk for having CKDu was lower in the high altitude/ low WBGT temperature environment compared to the lower altitude/ higher WBGT temperature environment. Traditional agriculture in Indonesia is generally carried out using hands and several types of simple manual equipment. In traditional agriculture practices, farmers use hoes to cultivate the soil, plant seeds into the soil manually using their hands and use a machete to harvest rice. This requires more physical activity and a higher physical workload compared to agriculture practices that use modern mechanical equipment. A study by Groborz and Juliszewski showed that the workload during the tasks assisted by mechanization was usually very low—or relatively low—and assisting work with mechanical devices reduces the workload of farmers [35]. The higher workload is postulated to have consequences for kidney health since strenuous physical activity in hot, humid climates can produce heat stress and dehydration which are considered as repeated risk factors in acute kidney injury episodes that could lead to CKD [36,37]. From our findings, we suggested that the type of farming may be a surrogate for workload and therefore perhaps a more important risk factor for CKDu than the elevation and WBGT differences seen in this study.

Other occupational factors that we analyzed in this study included exposure to agrochemicals. We found that the risk of CKDu increased along with the years of insecticide use. Adjusted by farm location, herbs consumption, BMI and the number of years work as a farmer, the risk of having CKDu among farmers who used insecticide 10–19 years and more than 20 years were 2.2 and 2.3 times higher compared to farmers who used insecticide less than 10 years. We also found that the risk of CKDu increased along with the increasing frequency of insecticide use. Adjusted by farm location, type of farming, BMI and the number of years work as a farmer, the risk of having CKDu among farmers who used insecticide 2–4 times per month, and more than four times per month were 1.2 and 2.3 times higher compared to farmers who used insecticide less frequent (2–4 times per planting season). For rice production in Indonesia, one planting season takes time around three to four months. A review study by Valcke et al. [11] reported that four studies with stronger designs and exposure assessment found an association between different pesticides and CKD/CKDu. We suspect that in Indonesia there is significant pesticide overuse, incorrect methods of handling and storage and also relatively less-educated farmers who used less mechanized forms of agriculture, increasing direct contact with agrochemicals [11]. Some of the nephrotoxic agents in pesticides include cadmium, arsenic, chromium and other heavy metals. Chronic exposure to those heavy metals is associated with chronic tubulointerstitial nephritis. The mechanism of the damage begins when the heavy metals accumulate in proximal tubule cells, causing both functional and structural damage, and resulted in reabsorptive and secretory defects. This process may involve local oxidative stress with associated lipid peroxidation, apoptosis and necrosis as common phenomena in the course of nephrotoxicity of these metals [38,39].

Limitations of our study include the method used in gathering information related to pesticide use in farming. We collected information on agrochemical exposures by questionnaire. We intended to calculate the intensity level of pesticide exposure using the general algorithm by Dosemeci et al [33]. However, this proved difficult because most farmers used so many brand names of pesticides: 122 brand names of insecticide, 17 brand names of fungicide, 5 brand names of herbicide with about 52% of those brand names not found in the registered agricultural pesticide list (could be a local brand name or could be the result of mispronounced/misspelled pesticide names by the farmers and/or enumerators). As a result, we focused our analysis on the insecticide used for the longest period and the most frequently to calculate the intensity level. Thus, the pesticide intensity scores and frequencies may be underestimated. Nevertheless, Valcke et al. concluded that existing studies in many countries had poor pesticide exposure assessment, and there was a need for future research on the assessment of lifetime exposures to relevant specific pesticides concerning CKD and CKDu [11].

Overall, based on our study results, the environmental and occupational risk factors of CKDu may not be limited to the farming methods and exposure of insecticides. There were many potential environmental and occupational risk factors that we have not examined in this study. Thus, it is necessary to conduct further research to investigate other potential environmental and occupational risk factors of CKDu.

Due to our findings that farmers in higher altitude/ lower WBGT locations were at higher risk for CKDu compared to farmers in lower altitude/ higher WBGT locations, there was a need to make a recommendation for future interventions in this high-risk population to improve its quality of life and renal function. The interventions that we recommend are encourage the farmers to visit local public health center routinely to check their health, including blood pressure and heart rate measurement [40,41]; and for health workers at the public health center, we recommend to do self-management support interventions on farmers with CKD/CKDu to improve self-care activity of the farmers [42].

## 5. Conclusions

We found that farmers in higher altitude/ lower WBGT locations were at higher risk for CKDu compared to farmers in lower altitude/ higher WBGT locations. However, once the type of farming was included (degree of mechanization) the findings become more explainable, that farmers who applied less mechanized type of farming, the risk for having CKDu was higher in the high altitude/ low WBGT locations compared to the lower altitude/ higher WBGT locations.

We also found that there was a statistically insignificant increase in the risk of CKDu with the longer lifetime duration of insecticide use and the more frequent use of insecticides, after controlling for other covariates including farm location.

We suggested that the type of farming and insecticide exposure may be important to be considered as a potential risk factor for CKDu, so there was a need to conduct future research to investigate more on the association of those factors with CKDu.

## Figures and Tables

**Table 1:** Demographic factors of male farmers in Karawang and Bogor in West Java.

Variables	Karawang ^1^ (*n* = 186) *n* (%)	Bogor ^2^ (*n* = 168) *n* (%)	Total
Age			
≥50	104 (55.9)	93 (55.4)	197
<50	82 (44.1)	75 (44.6)	157
Educational level			
Low	167 (89.8)	158 (94.0)	325
High	19 (10.2)	10 (6.0)	29
Diabetes mellitus			
Yes	4 (2.2)	2 (1.2)	6
No	182 (97.8)	166 (98.8)	348
Hypertension			
Yes	20 (10.8)	25 (14.9)	45
No	166 (89.2)	143 (85.1)	309
Blood pressure			
≥140/ ≥90	52 (28.0)	26 (15.5)	78
120–139/80–89	114 (61.3)	108 (64.3)	222
<120/<80	20 (10.8)	34 (20.2)	54
Blood sugar			
High (>200 mg/dL)	6 (3.2)	3 (1.8)	9
Normal	180 (96.8)	165 (98.2)	345

^1^ Karawang: location with low altitude and high WBGT. ^2^ Bogor: location with high altitude and low WBGT.

**Table 2:** Chronic kidney disease (CKD) parameters of male farmers in Karawang and Bogor, West Java.

Variables	Total *n* (%)	Karawang ^1^ (*n* = 186) *n* (%)	Bogor ^2^ (*n* = 168) *n* (%)
Serum Creatinine			
High	11 (3.1)	4 (2.2)	7 (4.2)
Normal	343 (96.9)	182 (97.8)	161 (95.8)
Proteinuria			
Positive (+2 or more)	15 (4.2)	7 (3.8)	8 (4.8)
Negative (0 or +1)	339 (95.8)	179 (96.2)	160 (95.2)
eGFR-MDRD			
<60 mL/min/1.73 m^2^	12 (3.4)	5 (2.7)	7 (4.2)
≥60 mL/min/1.73 m^2^	342 (96.6)	181 (97.3)	161 (95.8)
CKD Stages			
Stage 4 (eGFR15–29 mL/min/1.73 m^2^)	2 (0.6)	1 (0.5)	1 (0.6)
Stage 3 (eGFR30–59 mL/min/1.73 m^2^)	10 (2.8)	4 (2.2)	6 (3.6)
Stage 2 (eGFR60–89 mL/min/1.73 m^2^ & positive proteinuria)	69 (19.5)	27 (14.5)	42 (25.0)
Stage 1 (eGFR≥90 mL/min/1.73 m^2^ & positive proteinuria)	7 (2.0)	3 (1.6)	4 (2.4)
Normal (eGFR≥90 mL/min/1.73 m^2^ & negative proteinuria)	266 (75.1)	151 (81.2)	115 (68.5)
Any CKD			
Yes (Stage 1–4)	88 (24.9)	35 (18.8)	53 (31.5)
No (Normal)	266 (75.1)	151 (81.2)	115 (68.5)
CKD Status (subset of Any CKD above)			
CKDu *	66 (18.6)	26 (14.0)	40 (23.8)
CKD **	22 (6.3)	9 (4.8)	13 (7.7)
No CKD ***	266 (75.1)	151 (81.2)	115 (68.5)

^1^ Karawang: location with low altitude and high WBGT. ^2^ Bogor: location with high altitude and low WBGT. * CKDu included subjects with CKD stages 1–4, but excluded subjects with diabetes based on blood sugar concentration (>200 mg/dL considered as diabetes) or self-report diabetes or hypertension based on blood pressure (systole/diastole ≥140/ ≥90 considered as hypertension) or self-reported hypertension with medication or self-reported urethritis, gout and kidney stone. ** CKD included subjects with CKD Stages 1–4 without the exclusion of subjects with diabetes, hypertension, urethritis, gout, kidney stone. *** No CKD included subjects with Normal status of CKD stages.

**Table 3:** Distribution of male farmers in West Java according to CKD status (chronic kidney disease of unknown etiology (CKDu), CKD and No CKD), individual factors, occupational factors and environmental factors.

Variables	Total *n* (%)	CKDu * (*n* = 66) *n* (%)	CKD ** (*n* = 22) *n* (%)	No CKD *** (*n* = 266) *n* (%)
Age				
≥50	197 (55.6)	38 (57.6)	11 (50.0)	148 (55.4)
<50	157 (44.4)	28 (42.4)	11 (50.0)	118 (44.6)
Years of work as farmer				
≥20 years	204 (57.6)	39 (59.1)	13 (59.1)	152 (57.1)
15–19 years	43 (12.2)	6 (9.1)	2 (9.1)	35 (13.2)
<15 years	107 (30.2)	21 (31.8)	7 (31.8)	79 (29.7)
Type of farming				
Traditional	118 (33.3)	25 (37.9)	7 (31.8)	86 (32.3)
Mix (traditional and modern)	193 (54.5)	37 (56.1)	13 (59.1)	143 (53.8)
Modern	43 (12.2)	4 (6.0)	2 (9.1)	37 (13.9)
Work as farmer per day				
≥8 h	117 (33.0)	20 (30.3)	4 (18.2)	93 (35.0)
6–7 h	126 (35.6)	30 (45.5)	9 (40.9)	87 (32.7)
≤5 h	111 (31.4)	16 (24.2)	9 (40.9)	86 (32.3)
BMI (*n* = 343)				
25.0– ≥30.0	54 (15.7)	6 (9.1)	6 (27.3)	42 (16.3)
<18.5–24.9	289 (84.3)	58 (90.9)	16 (72.7)	215 (83.7)
Kidney stone				
Yes	4 (1.1)	1 (1.5)	1 (4.5)	2 (0.8)
No	350 (98.9)	65 (98.5)	21 (95.5)	264 (99.2)
Urethritis (≥3 times in last 3 months)				
Yes	17 (4.8)	4 (6.1)	1 (4.5)	12 (4.5)
No	337 (95.2)	62 (93.9)	21 (95.5)	254 (95.5)
Family history of kidney disease				
Yes	9 (2.5)	1 (1.5)	0 (0)	8 (3.0)
No	345 (97.5)	65 (98.5)	22 (100)	258 (97.0)
Herbs (traditional drinks)				
Yes	86 (24.3)	17 (25.8)	9 (40.9)	60 (22.6)
No	268 (75.7)	49 (74.2)	13 (59.1)	206 (77.4)
Alcohol consumption				
Current	9 (2.5)	0 (0)	1 (4.5)	8 (3.0)
Past or never	345 (97.5)	66 (100)	21 (95.5)	258 (97.0)
Smoking				
Current	288 (81.4)	54 (81.8)	17 (77.3)	217 (81.6)
Past or never	66 (18.6)	12 (18.2)	5 (22.7)	49 (18.4)
Drugs consumption ****				
Yes	135 (38.1)	28 (42.4)	8 (36.4)	99 (37.2)
No	219 (61.9)	38 (57.6)	14 (63.6)	167 (62.8)
Water source for drinking				
Ground well/spring water	204 (57.6)	41 (62.1)	16 (72.7)	147 (55.3)
Surface water/pond/river	11 (3.1)	3 (4.5)	1 (4.5)	7 (2.6)
Government piped/bottled	139 (39.3)	22 (33.4)	5 (22.8)	112 (42.1)
Farm location				
Karawang ^1^ (low altitude/high WBGT)	186 (52.5)	26 (39.4)	9 (40.9)	151 (56.8)
Bogor ^2^ (high altitude/low WBGT)	168 (47.5)	40 (60.6)	13 (59.1)	115 (43.2)

^1^ Karawang: location with low altitude and high WBGT. ^2^ Bogor: locationwith high altitude and low WBGT. * CKDu included subjects with CKD stages 1–4, but excluded subjects with diabetes based on blood sugar concentration (>200 mg/dL considered as diabetes) or self-reported diabetes or hypertension based on blood pressure (systole/diastole ≥140/ ≥90 considered as hypertension) or self-reported hypertension with medication or self-reporting of urethritis, gout and kidney stone. ** CKD included subjects with CKD stages 1–4 without the exclusion of subjects with diabetes, hypertension, urethritis, gout, kidney stone. *** No CKD included subjects with Normal status of CKD stages. **** NSAID drug consumption in the last 3 months.

**Table 4:** Pesticide use and CKD/CKDu among male farmers in study location in West Java.

Variables	Total	CKDu (*n* = 66) *n* (%)	CKD (*n* = 22) *n* (%)	No CKD (*n* = 266) *n* (%)
Pesticide use				
Yes	334 (94.4)	60 (90.9)	20 (90.9)	254 (95.5)
No	20 (5.6)	6 (9.1)	2 (9.1)	12 (4.5)
Maximum years of any insecticide used (*n* = 334)				
≥20 years	137 (41.0)	23 (38.3)	8 (36.4)	106 (41.7)
10–19 years	97 (29.1)	13 (21.7)	6 (31.8)	78 (30.7)
<10 years	100 (29.9)	24 (40.0)	6 (31.8)	70 (27.6)
Maximum frequency of any insecticide currently used (*n* = 334)				
More than 4 times per month	45 (13.5)	5 (8.3)	0 (0)	40 (15.7)
2–4 times per month	149 (44.6)	23 (38.3)	8 (40.0)	118 (46.5)
2–4 times per planting season	140 (41.9)	32 (53.4)	12 (60.0)	96 (37.8)
Maximum intensity level of current insecticide exposure *				
≥16.0	207 (58.5)	40 (60.6)	14 (63.6)	153 (57.5)
0–15.9	147 (41.5)	26 (39.4)	8 (36.4)	113 (42.5)
Insecticide use at home				
Yes	221 (62.4)	38 (57.6)	10 (45.4)	173 (65.0)
No	133 (37.6)	28 (42.4)	12 (54.6)	93 (35.0)

* Median intensity level of current insecticide exposure = 16.0.

**Table 5:** CKDu among male farmers * according to farm location in West Java and stratified by type of farming.

Risk Factors	CKDu		Total	POR	95% CI	*p*-Value
	Yes	No				
	*n* (%)	*n* (%)				
Location (*n* = 332)						
High altitude and low WBGT	40 (60.6)	115 (43.2)	155	2.0	1.2–3.5	0.01
Low altitude and high WBGT	26 (39.4)	151 (56.8)	177	Reference		
Traditional type of farming (*n* = 111)						
High altitude & low WBGT location	24 (96.0)	76 (88.4)	100	3.2	0.4–25.9	0.29
Low altitude & high WBGT location	1 (4.0)	10 (11.6)	11	Reference		
Mixed type of farming (*n* = 180)						
High altitude & low WBGT location	15 (40.5)	27 (18.9)	42	2.9	1.3–6.4	0.01
Low altitude & high WBGT location	22 (59.5)	116 (81.1)	138	Reference		
Modern type of farming (*n* = 41)						
High altitude & low WBGT location	1 (25.0)	12 (32.4)	13	0.7	0.1–7.4	0.76
Low altitude & high WBGT location	3 (75.0)	25 (67.6)	28	Reference		

* Subjects with CKD were excluded. POR: Prevalence Odds Ratio.

**Table 6:** Unadjusted and adjusted odds of CKDu according to insecticide use among male farmers in study location in West Java.

Insecticide Use	Total *n*	CKDu *n* (column%)	Unadjusted POR (95% CI)	Adjusted POR (95% CI)
Maximum years of any insecticide used ^1^				
≥20 years	129	23 (38.3)	0.6 (0.3–1.2)	2.3 (0.9–5.6)
10–19 years	91	13 (21.7)	0.5 (0.2–1.0)	2.2 (0.9–5.2)
<10 years	94	24 (40.0)	Reference	Reference
Maximum frequency of any insecticide currently used ^2^				
>4x/month	45	5 (8.3)	0.4 (0.1–1.0)	2.3 (0.6–9.6)
2–4x/month	149	23 (38.3)	0.6 (0.3–1.1)	1.2 (0.5–2.9)
2–4x/planting season	140	32 (53.4)	Reference	Reference

^1^ Adjusted by: farm location, herbs consumption, BMI number of years work as farmer. ^2^ Adjusted by: farm location, type of farming, BMI, number of years work as farmer.

## References

[1] United State Renal Data System US Renal Data System 2019 Annual Data Report: Epidemiology of Kidney Disease in the United States. www.usrds.org/2019/view/USRDS_2019_ES_final.

[2] Jha V., Garcia G.G., Iseki K., Li Z., Naicker S., Plattner B., Saran R., Wang A.Y.-M. (2013). Chronic kidney disease: Global dimension and perspectives. Lancet.

[3] Peraza S., Wesseling C., Aragon A., Leiva R., Trabanino R.A.G., Torres C., Jakobsson K., Elinder C.G., Hogstedt C. (2012). Decreased Kidney Function Among Agricultural Workers in El Salvador. Am. J. Kidney Dis..

[4] Almaguer M., Herrera R., Orantes C.M. (2014). Chronic Kidney Disease of Unknown Etiology in Agricultural Communities. MEDICC Rev..

[5] Hill N.R., Fatoba S.T., Oke J.L., Hirst J.A., O’Callaghan C.A., Lasserson D.S. (2016). Global Prevalence of Chronic Kidney Disease—A Systematic Review and Meta-Analysis. PLoS ONE.

[6] Hooi L.S., Ong L.M., Ahmad G., Bavanandan S., Ahmad N.A., Naidu B.M., Mohamud W.M.W., Yusoff M.F.M. (2013). A population-based study measuring the prevalence of chronic kidney disease among adults in West Malaysia. Kidney Int..

[7] Indonesia Ministry of Health (2013). Basic Health Research—Riskesdas 2013.

[8] Indonesian Renal Registry 10th Report of Indonesian Renal Registry—2017. https://www.indonesianrenalregistry.org.

[9] Weaver V.M., Fadrowski J.J., Jaar B.G. (2015). Global dimensions of chronic kidney disease of unknown etiology (CKDu): A modern era environmental and/or occupational nephropathy?. BMC Nephrol..

[10] Jayasinghe S. (2014). Chronic kidney disease of unknown etiology should be renamed chronic agrochemical nephropathy. MEDICC Rev..

[11] Valcke M., Levasseur M.E., da Silva A.S., Wesseling C. (2017). Pesticide exposures and chronic kidney disease of unknown etiology: An epidemiologic review. Environ. Health.

[12] Statistic Indonesia. https://www.bps.go.id/publication/2014/05/05/statistic.

[13] Indonesia Ministry of Agriculture (2015). Statistik Prasarana dan Sarana Pertanian Tahun 2011–2015 (Agricultural Infrastructure and Facilities Statistic 2011–2015).

[14] Faqih A., Boer R., Soeparno H., Pasandaran E., Syarwani M., Dariah A., Pasaribu S.M., Saad N.S. (2013). Fenomena Perubahan Iklim di Indonesia. Politik Pembangunan Pertanian Dalam Menghadapi Perubahan Iklim.

[15] Lucas R.A., Epstein Y., Kjellstrom T. (2014). Excessive occupational heat exposure: A significant ergonomic challenge and health risk for current and future workers. Extrem. Physiol. Med..

[16] Trihono P.P., Rhodia L., Karyanti M.R. (2018). Kidney Disease Profiles among Adolescents in Indonesia. Acta Med. Indones..

[17] Prodjosudjadi W., Suhardjono A. (2009). End-stage renal disease in Indonesia: Treatment development. Ethn. Dis..

[18] Prodjosudjadi W., Suwitra K., Widiana I.G.R., Loekman J.S., Nainggolan G., Prasanto H., Wijayanti Y., Sja’Bani M., Nasution M.Y., Basuki W. (2009). Detection and prevention of chronic kidney disease in Indonesia: Initial community screening. Nephrology.

[19] Hidayati E.L., Trihono P.P. (2011). Admission characteristics of pediatric chronic kidney disease. Paediatr. Indones..

[20] Farida L.S., Thaha M., Susanti D. (2018). Characteristics of patients with end-stage renal disease at Dialysis Unit Dr. Soetomo General Hospital Surabaya. Biomol. Health Sci. J..

[21] BPS-Statistic of Karawang Regency (2018). Kabupaten Karawang Dalam Angka 2018 (Karawang Regency in Figures 2018).

[22] BPS-Statistic of Bogor Regency (2018). Kabupaten Bogor Dalam Angka 2018 (Bogor Regency in Figures 2018).

[23] STROBE Statement—Strengthening the Reporting of Observational Studies in Epidemiology. https://www.strobe-statement.org/index.php?id=available-checklists.

[24] Caplin B., Jakobsson K., Glaser J., Nitsch D., Jha V., Singh A., Correa-Rotter R., Pearce N. (2017). International collaboration for the epidemiology of eGFR in low and middle income populations—Rationale and core protocol for the disadvantaged populations eGFR Epidemiology Study (DEGREE). BMC Nephrol..

[25] Riley L., Guthold R., Cowan M., Savin S., Bhatti L., Armstrong T., Bonita R. (2016). The World Health OrganizationSTEPwise Approach to Noncommunicable Disease Risk-Factor Surveillance: Methods, Challenges, and Opportunities. Am. J. Public Health.

[26] World Health Organization The STEPS Instrument and Support Materials. http://www.who.int/chp/steps/instrument/en/.

[27] Prodia. http://www.prodia.co.id/en.

[28] Ogawa J., Nirdnoy W., Tabata M., Yamada H., Shimizu S. (1995). A new enzymatic method for the measurement of creatinine involving a novel ATP-dependent enzyme, N-methylhydantoin amidohydrolase. Bioschi. Biotech. Biochem..

[29] Zawada R.J.X., Kwan P., Olszewski K.L., Llinas M., Huang S.-G. (2009). Quantitative determination of urea concentrations in cell culture medium. Biochem. Cell Biol..

[30] Michels W.M., Grootendorst D.C., Verduijn M., Elliott E.G., Dekker F.W., Krediet R.T. (2010). Performance of the Cockcroft-Gault, MDRD, and New CKD-EPI Formulas in Relation to GFR, Age, and Body Size. Clin. J. Am. Soc. Nephrol..

[31] Combur-Test^®^ Strip—Diagnostics Roche. https://diagnostics.roche.com/global/en/products/instruments/combur_chemstripnephurnitur.html.

[32] Ramdhan D.H., Prihartono N.A., Fitria L., Wahyono T.Y.M., Kongtip P., Woskie S. (2020). Health problem and work practices among male rice farmers from different altitude areas in Karawang and Bogor, West Java, Indonesia. Int. J. Occup. Environ. Health.

[33] Dosemeci M., Alavanja M.C.R., Rowland A.S., Mage D., Zahm S.H., Rothman N., Lubin J.H., Hoppin J.A., Sandler D.P., Blair A. (2002). A quantitative approach for estimating exposure to pesticides in the agricultural health study. Ann. Occup. Hyg..

[34] Rajapakse S., Shivanthan M.C., Selvarajah M. (2016). Chronic kidney disease of unknown etiology in Sri Lanka. Int. J. Occup. Environ. Health.

[35] Groborz A., Juliszewski T. (2013). Comparison of farmers workload by manual and mechanical tasks on family farms. Ann. Agric. Environ. Med..

[36] Nerbass F.B., Pecoits-Filho R., Clark W.F., Sontrop J.M., McIntyre C.W., Moist L. (2017). Occupational heat stress and kidney health: From farms to factories. Kidney Int. Rep..

[37] García-Trabanino R., Jarquín E., Wesseling C., Johnson R.J., González-Quiroz M., Weiss I., Glaser J., José V.J., Stockfelt L., Roncal C. (2015). Heatstress, dehydration, and kidney function in sugarcane cutters in El Salvador—A cross-shift study of workers at risk of Mesoamerican nephropathy. Environ. Res..

[38] Brook D. (2009). Final Scoping Study Report—Epidemiology of Chronic Kidney Disease in Nicaragua.

[39] Sabolic I. (2006). Common mechanisms in nephropathy induced by toxic metals. Nephron Physiol..

[40] Mazoteras-Pardo V., Becerro-De-Bengoa-Vallejo R., Losa-Iglesias M.E., López-López D., Rodríguez-Sanz D., Casado-Hernández I., Calvo-Lobo C., Palomo-López P. (2019). QardioArm Upper Arm Blood Pressure Monitor Against Omron M3 Upper Arm Blood Pressure Monitor in Patients With Chronic Kidney Disease: A Validation Study According to the European Society of Hypertension International Protocol Revision. J. Med. Internet Res..

[41] Mazoteras-Pardo V., Becerro-De-Bengoa-Vallejo R., Losa-Iglesias M.E., López-López D., Calvo-Lobo C., Rodríguez-Sanz D., Eva María Martínez-Jiménez E.M., Palomo-López P. (2020). An Automated Blood Pressure Display for Self-Measurement in Patients with Chronic Kidney Disease (iHealth Track): Device Validation Study. JMIR Mhealth Uhealth.

[42] Zimbudzi E., Lo C., Misso M.L., Ranasinha S., Kerr P.G., Teede H.J., Zoungas S. (2018). Effectiveness of self-management support interventions for people with comorbid diabetes and chronic kidney disease: A systematic review and meta-analysis. Syst. Rev..

